# TreeJ: an ImageJ plugin for interactive cell lineage reconstruction from static images

**DOI:** 10.1186/s13007-023-01106-x

**Published:** 2023-11-16

**Authors:** Elise Laruelle, Jean-Christophe Palauqui, Philippe Andrey, Alain Trubuil

**Affiliations:** 1 Université Paris-Saclay, INRAE, AgroParisTech, Institut Jean-Pierre Bourgin (IJPB), Route de Saint Cyr, 78000 Versailles, France; 2 MaIAGE, INRAE, Université Paris-Saclay, Domaine de Vilvert, 78350 Jouy-en-josas, France; 3 Sainsbury Laboratory, Cambridge University, Bateman Street, CB2 1LR Cambridge, UK

**Keywords:** Cell lineage, Image annotation, ImageJ software

## Abstract

**Background:**

With the emergence of deep-learning methods, tools are needed to capture and standardize image annotations made by experimentalists. In developmental biology, cell lineages are generally reconstructed from time-lapse data. However, some tissues need to be fixed to be accessible or to improve the staining. In this case, classical software do not offer the possibility of generating any lineage. Because of their rigid cell walls, plants present the advantage of keeping traces of the cell division history over successive generations in the cell patterns. To record this information despite having only a static image, dedicated tools are required.

**Results:**

We developed an interface to assist users in the building and editing of a lineage tree from a 3D labeled image. Each cell within the tree can be tagged. From the created tree, cells of a sub-tree or cells sharing the same tag can be extracted. The tree can be exported in a format compatible with dedicated software for advanced graph visualization and manipulation.

**Conclusions:**

The TreeJ plugin for ImageJ/Fiji allows the user to generate and manipulate a lineage tree structure. The tree is compatible with other software to analyze the tree organization at the graphical level and at the cell pattern level. The code source is available at https://github.com/L-EL/TreeJ.

## Background

Understanding how cell organizations emerge is one of the major challenges in developmental biology. Cells at any particular stage result from several rounds of cell divisions. Reconstructing the history of successive cell division events as a cell lineage tree is thus essential for understanding cell patterning [1–3]. In situations amenable to time-lapse microscopy, various programs exist to track cells and their divisions (ALT [3], LineageTracker [4], MorphographX [5], etc.). However, in some contexts, tissue fixation is required prior to image acquisition. In plants, because of the absence of cell migration, cell lineage can still be inferred from the resulting static images, by recursively backtracking cell division events. This requires that the patterning is stereotyped enough or that clues are available for the biologist to identify daughter cells. One of the clues is the cell geometry that can be conserved during tissue growth. An expert eye carries this knowledge that can be difficult to translate into an algorithm [6].

With the emergence of deep learning methods, neural networks will increasingly be used to predict lineages, as in other biological kingdoms [7], but more manually annotated data will be needed to train the networks. In addition, no user-friendly tool has been proposed to date in the bioimage informatics community to assist biologists in reconstructing cell lineages from static images [6].

Here, we present a graphical interface integrated in ImageJ/Fiji [8–10] to reconstruct a lineage tree from a static 3D image stack. The interface design is intuitive and the experimenter can easily link and annotate cells in a recursive way.

## Implementation

The aim of this work was to develop a tool for building interactively a lineage tree from a labeled image of cells in a developing tissue or organism. To conceive this tool, we choose ImageJ/Fiji, a user-friendly software that is widely used by experimental scientists. ImageJ/Fiji contains a plugin development functionality. We developed the TreeJ plugin in Java within this framework.

**Fig. 1:**
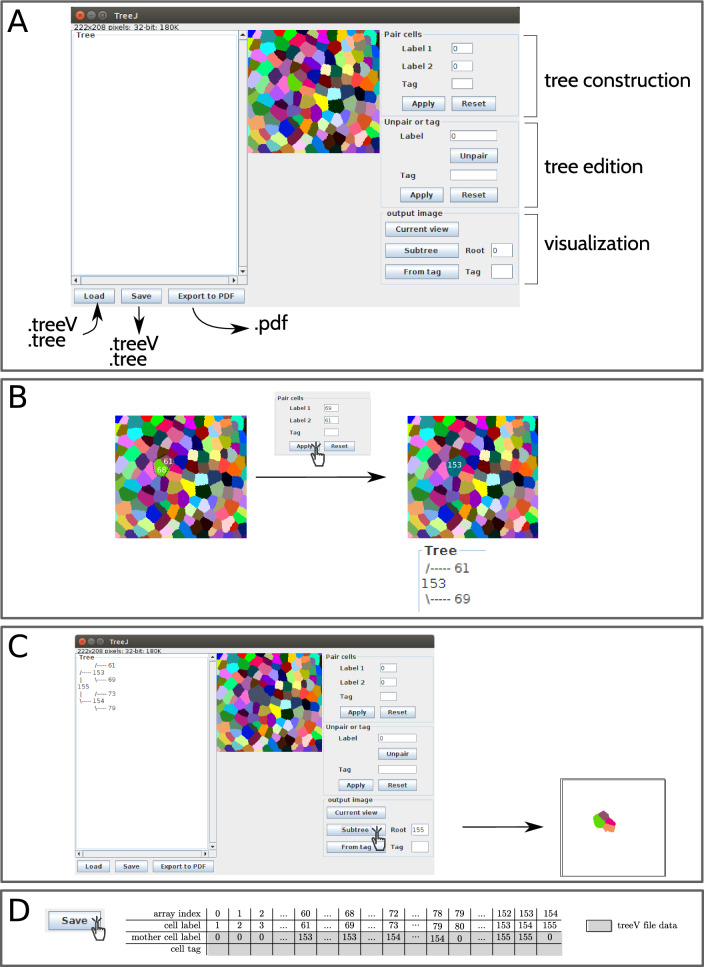
TreeJ interface and main user interactions.** A** TreeJ interface showing the surface of an *A. thaliana* shoot apical meristem.** B** Binding of sister cells, the elementary step during the reconstruction of a cell lineage tree.** C** Pulling out a set of sister cells from their common ancestor node.** D** Saving the tree built in C using the treeV format. The treeV format contains 2 arrays (shown in gray) storing tree structure and annotations, respectively. In this example, the second array is empty, because the tree is not annotated

**Fig. 2:**
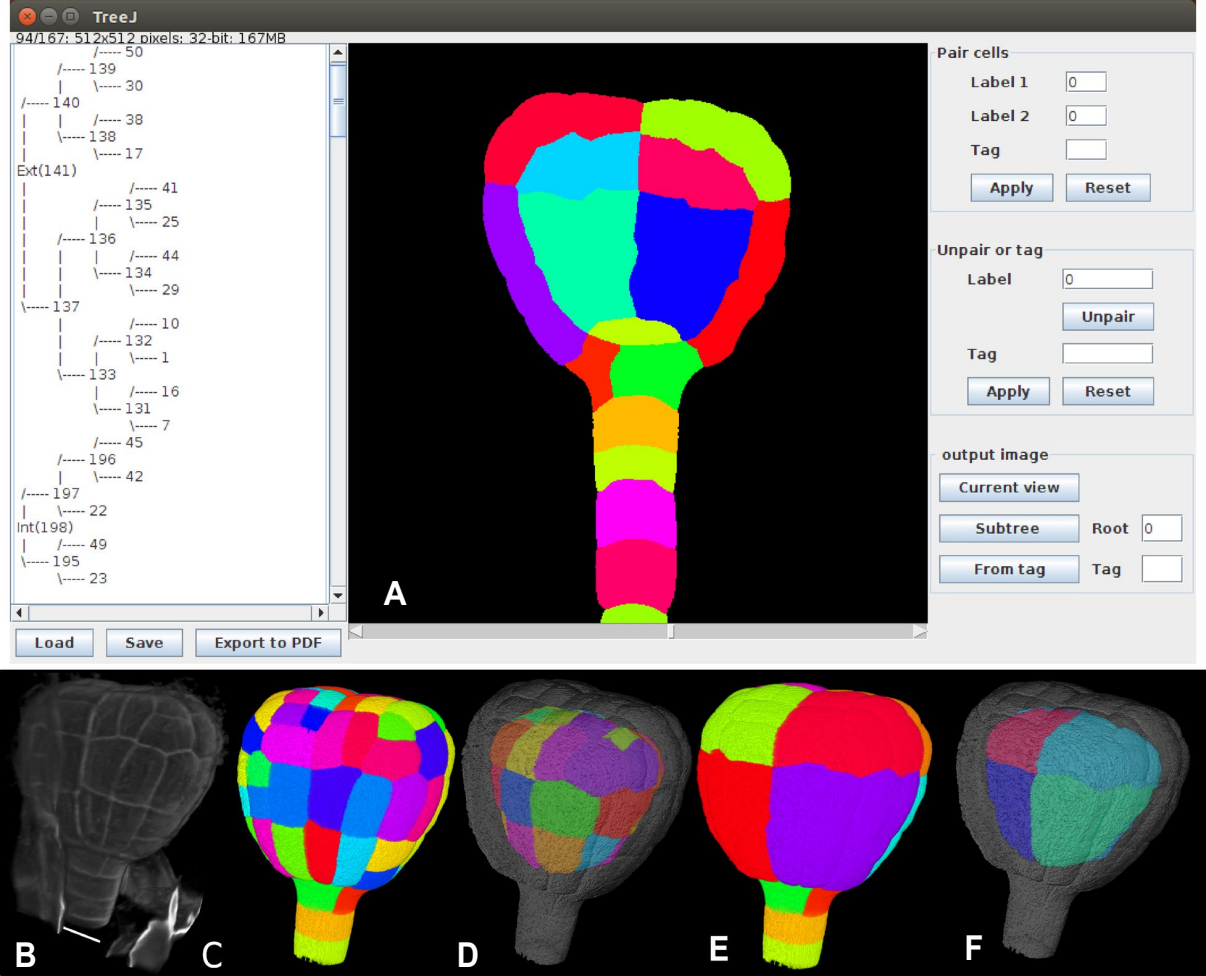
Cell lineage reconstruction and annotation using TreeJ: illustration with a plant embryo. **A** TreeJ interface showing an *A. thaliana* 122-cells embryo tracked back over four cell generations.** B** 3D embryo raw image (scale bar: 10 $\mu m$). **C** 3D segmentation of image shown in **B**. **D** Output image of tagged internal tree nodes. **E** Output image after the back tracking, external parts. **F** Output image after the back tracking, inner parts. 3D renderings were made using the ImageJ 3D viewer [11]

### GUI

The created plugin embeds the current image in a graphical interface (Fig. 1A). The TreeJ interface is composed of three parts. The 2D image, or the current slice of the 3D image stack, is shown in the middle to allow direct point-and-click interactions for selecting the cells to link. The right-panel displays buttons to construct, label, and extract trees and sub-trees. The left-panel frame displays the ongoing tree and provides input/output buttons. Depending on the user question, different outputs can be generated.

### Input

TreeJ is compatible with a large spectrum of labeled images obtained by any region segmentation software. The interface can be launched on any 2D or 3D label image from 8 to 32 bits. The labeling can be discontinuous (i.e., missing labels), as frequently encountered as a result of post-processing operations applied to segmented images. The only assumption on the input image is that the segmentation labels are strictly positive; if present, the label 0 should be the background of the segmented structure (limitation explained in the ’Tree file’ paragraph below).

### Tree underlying structure

Internally, TreeJ constructs binary tree structures from the user input directives. Java classes have been used to organize the tree(s) with node objects storing cell information. A node is composed of a cell label (from 0 to the higher label number $label_{max}$), a user annotation (initialized with an empty string) and the labels of the two daughter cells (initialized to none for the non-mother cells present in the input segmentation, i.e., leaves of the tree).

Upon plugin opening, cells (i.e. labels) present in the image generate leaves (nodes without linked daughter cells). Then, new nodes can be created by the user assignment of cell links (Fig. 1B). A new cell label is assigned to each newly created mother cell. This label is automatically defined as $label_{max}+1$ and attached to the created node. The new node stores also the labels of the linked cells (i.e., the daughter cells). This process progressively builds a tree (or several disjoint trees), which is displayed in the left panel of the interface (Fig. 1C).

### Tree file

TreeJ allows to save and load a reconstructed tree in an home made format called treeV (file extension .treeV). A treeV file is an ASCII file, containing two arrays: one encoding the tree and the other encoding the annotations of each cell. For an embryo containing *n* cells, the initial structure (i.e., without lineage) of the treeV will be:
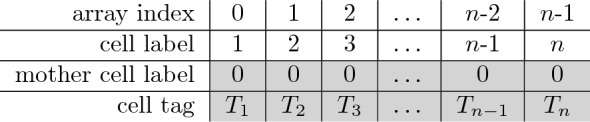


where the two gray lines are the treeV data and $T_{i}$ is the tag of the *i*-th cell.

The tree(s) is stored as a list with as many elements as the highest label number, typically corresponding to the number of input cells (*n* in the table above) added to the number of reconstructed mother cells (Fig. 1D). By matching the indexes of the list and the labels of the cell, the tree(s) structure can be encoded in only one array. Each position in the array corresponds to a cell. The value stored in the array at any position gives the label of the corresponding mother cell.

For the root(s) of the constructed tree(s), the value 0 is assigned to indicate the absence of mother cell. This implies that TreeJ implicitly assumes that no cell is labeled 0 in the input segmentation. Otherwise, mismatch assignment would result.

The annotations are saved in the treeV file as an array of character strings indexed by cell labels. By default, the annotation of each cell is an empty string. TreeV files can be loaded by TreeJ to continue, edit or visualize a previous lineage reconstruction. To transfer the lineage in another software, Newick format is also available as saving format.

## Results: TreeJ functionalities

Experimentalist’s observations are sometimes untraceable by an algorithm and need to be saved and structured to be analyzed and shared. In plant epithelia, the lineage is one of these data that can be intuitively deduced from the cell pattern by the biologist. Tree data structures are available to represent and exploit such observations. TreeJ provides the interface between these structures and the experimentalist to generate, correct, annotate and visualize cell lineages.

### Lineage reconstruction

The user reconstructs the lineage tree by recursively pairing sister cells (Fig. 1B). To link two cells, the user must either click the pair of cells on the image or enter manually their label values in the dedicated fields. Validating the pairing results in a new node being added to the tree, the merging of the pair of cells, and the automatic update of the display.

Errors can easily be corrected at any depth in the tree. The unlink button undoes erroneous pairings by splitting back any selected mother cell.

**Fig. 3:**
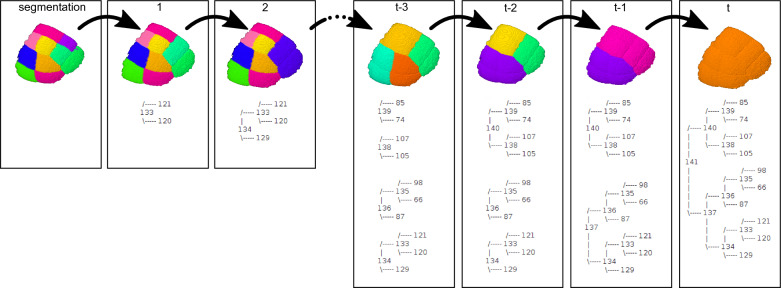
Cell lineage reconstruction in a protodermal domain. Combination of 3D visualizations of TreeJ outputs generated with the ImageJ 3D viewer [11], and of trees shown in the TreeJ frame during the backtracking process. Outputs obtained by recursively tracking cell divisions back to the progenitor cell of the considered domain at the 16-cells embryo stage. Backtracking containing t steps

### Annotating cells and sub-trees

TreeJ allows the introduction of biologically important information such as cell identity or localization in specific anatomical domains. Each node of the cell lineage tree can be tagged with an arbitrary text identifier. This annotation can be done when pairing cells, or later on. The same tag can be used for different cells (mother and daughters), thus supporting the annotation of whole domains (see the displayed tree in Fig. 2A).

### Output

At any time during the reconstruction of a lineage tree, the user can create an independent copy of the modified label image, which can thus be visualized and analyzed with any tool available in ImageJ (see examples developed in the applications below). TreeJ also allows to create images containing a subset of cells from a sub-tree specified by its mother cell label or a subset of cells sharing the same tag (Fig. 1C and Fig. 2D). This provides further opportunities for validating the reconstructed lineages and to analyze quantitatively selected anatomical domains.

The reconstructed tree graphs can be saved under PDF, treeV, or Newick file formats. TreeV, a home made format, can be taken as input by TreeJ, thus allowing lineage reconstruction and corrections over several sessions. Newick is a generic tree format recognized by other tools for further tree processing.

## Applications

### Lineage reconstruction of an *Arabidopsis thaliana* embryo

The *Arabidopsis thaliana* early embryo development is a stereotypical process with regard to geometry and patterning [12–14]. Starting from a unique cell, the embryo establishes the main tissues and organs in less than ten cell generations. Tissue patterning is an important aspect of embryo development, which appears very early in the process. At the third generation, an apical-basal axis is established and, at the fourth cell generation, the protoderm is formed from a periclinal division with an asymmetrical volume-ratio. After the 4th cell generation, in the 16-cells embryo, divisions start to be variable.

Because of the thickness of the seed coat, embryos must be fixed to study their cellular organization (note that it is possible to follow embryo development using live imaging [13], but the resolution is not accurate enough to properly segment cells in 3D). The use of fixed developmental times complicates the study of the cell pattern dynamics, but the lineage can still be inferred because of the cell walls that almost fix the cell organization. As an example, in order to follow the evolution of the volume asymmetry between protodermal and internal domains downstream the fourth generation, we considered an embryo of 122 cells (7th generation). Cell walls were stained with Propidium Iodide [15] (Fig. 2B). The acquired 3D image was segmented with the *Morphological Segmentation* ImageJ plugin [16](Fig. 2C). The segmentation was manually corrected when errors were present.

We used TreeJ to reconstruct lineage trees (Fig. 2A). Recursively, sister cells were inferred depending on the compatibility of their cell shapes, but also depending on the expert knowledge of embryo development (Fig. 3). Once the embryo had been tracked back to the 16-cells stage, we annotated the mother cells corresponding to the protoderm (Ext) and the inner tissues (Int) (Fig. 2A).

In the literature, it has been described that the cell divisions are almost synchronized during the early embryo development [17]. With the improvements in imaging methods, the time resolution has shown a delay between the cell divisions. This observation is well seen in older embryo lineage trees [12, 13]. Similarly, using TreeJ on a 122-cells embryo, the reconstructed lineage presented a cell generational gap. Unbalanced trees were observed in the reconstructed lineage (Fig. 2A and Fig. 3). The protoderm layer had at least one additional cell generation compared with internal tissues.

Using the tag image extraction functionality of TreeJ, we separated in two distinct images the cells descending from the ancestors of either the epidermal or the inner tissues (Fig. 2D). With the tools contained in ImageJ, we then analyzed the difference of volume and cell number between the two tissues. We extracted cell volumes in each image with the Analyze Regions 3D functionality of the MorphoLibJ package. We observed that more cells were present in the protoderm (73 cells) than in the inner tissue (49 cells). This result was consistent with the reported generational shift [13]. In addition, the average volume for internal cells was $468.8\pm 198.7\ \mu m^3$ (average ± s.d.) and $404.3\pm 140.3\ \mu m^3$ for external cells. These volume distributions were also in agreement with previous reports [12].

Using the current view image extraction functionality of TreeJ, we also obtained the volumes of the rewound cells at the fourth generation. We split the rewound cells from the four different quarters of embryo at generation 4 (with ImageJ tools for labeled images from [18]). We then computed the average volume ratio between protodermal cells and internal cells in the apical and in the basal domains of each quarter of the rewound 122-cells embryo. The average volume ratios in the apical and in the basal domains were $0.70\pm 0.06$ and $0.83\pm 0.04$, respectively. Thus, the volume asymmetry between the protoderm and the internal tissue [12, 14] is still present after more than two cell generations.

### Reconstructed dynamics of *Arabidopsis thaliana* embryo development

**Fig. 4:**
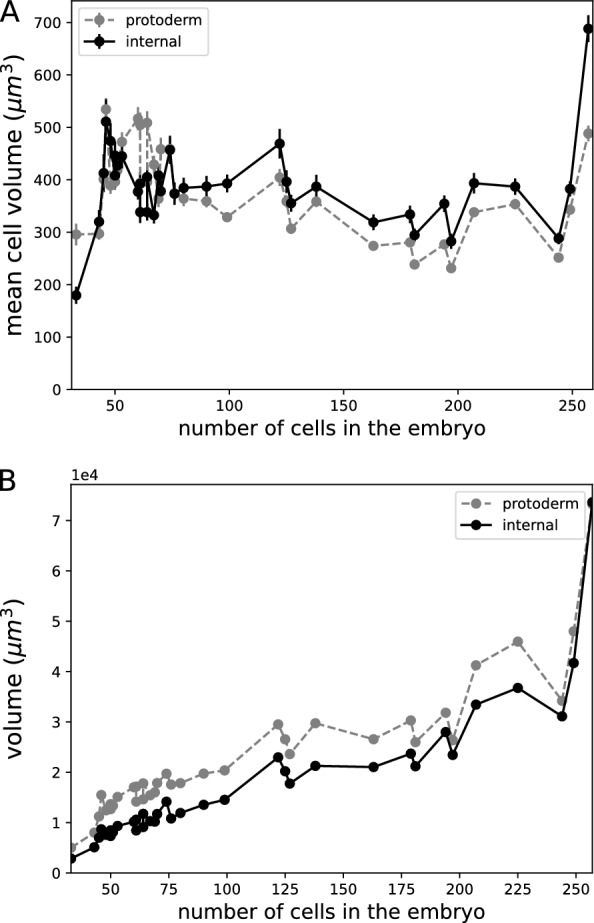
Dynamics of cell and domain growth during *Arabidopsis thaliana* embryo development. Measurements over 36 embryos (from [19])(dots) ordered by their total number of cells.** A** Growth dynamics for internal and for external (protoderm) cells. Error bars: s.e.m.** B** Growth dynamics of the internal and the external (protoderm) domain

Using TreeJ over several embryos, fixed at different stages of their development, allows to reconstruct an approximation of the embryogenesis dynamics [19]. We annotated the internal and protodermal mother cells of 36 embryos between 33 and 257 cells. Using a python script, annotations from TreeJ were used in combination with the segmented images to compute the distributions of cell volumes (Fig. 4A) and the volume of every tissue per embryo (Fig. 4B). Ordering embryos by their numbers of cells allowed us to reconstruct a temporal evolution of these quantities.

**Fig. 5:**
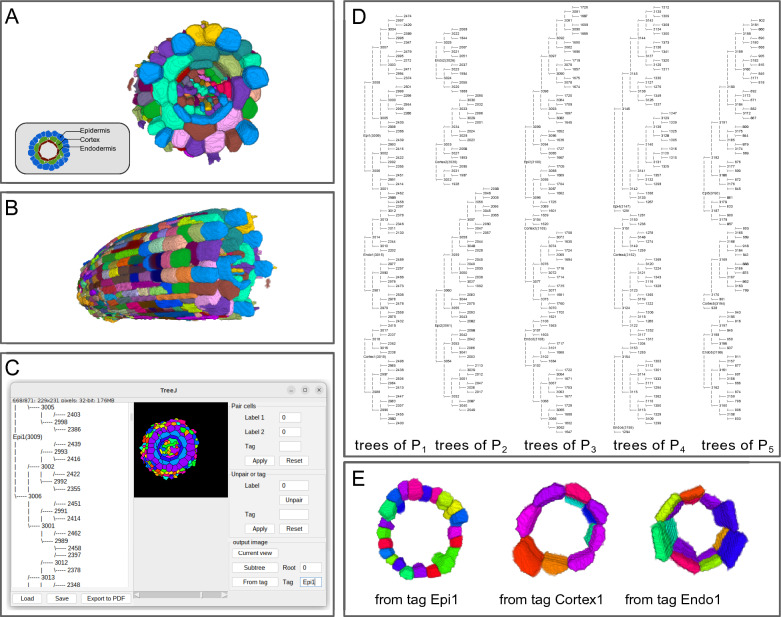
Grouping and annotations of cells in an *Arabidopsis thaliana* root.** A**,** B** 3D visualizations with the ImageJ 3D viewer [11] of the segmentation obtained with the *Morphological Segmentation* ImageJ plugin [16], top and side views, respectively.** C** TreeJ interface after tree reconstruction and tagging.** D** Reconstructed trees for each position ($P_{x}$ with *x* from 1 to 5) and each layer. Tags assigned to the grouped cells contain the layer name and the position number.** E** 3D visualizations with the ImageJ 3D viewer of the images obtained using tag-based selection of the three layers at Position 1 (top view)

A globally decreasing trend in the average cellular volume in the two domains was observed (Fig. 4A). This tendency is consistent with the relatively faster pace of cell proliferation compared to cell growth during early embryogenesis [14]. In spite of fluctuations in mean cell volume between successive times, due to variability between cells and between embryos, there was a marked difference in the average volume between cells in the protoderm and cells in the inner tissues. The higher volume in the internal tissues can hypothetically be explained by a lower number of cells, because of the cell generation delay. The higher total volume of the protoderm compared to the volume of inner tissue (Fig. 4B) is consistent with this interpretation.

These results illustrate how TreeJ can help to analyze and reveal global trends in the dynamics of different domains from fixed observations, thanks to the manual annotations that can be performed in addition to the reconstruction of cell lineages.

### Other application of TreeJ

TreeJ facilitates the reconstruction of trees by linking cells. The reason to link cells together can be due to a lineage relationship, but it can also be due to any other types of relation, as a spatial relation or a functional relation. In the example below we illustrate how TreeJ can also be used for the purpose of cell annotation and grouping, independently of any lineage relationship.

**Fig. 6:**
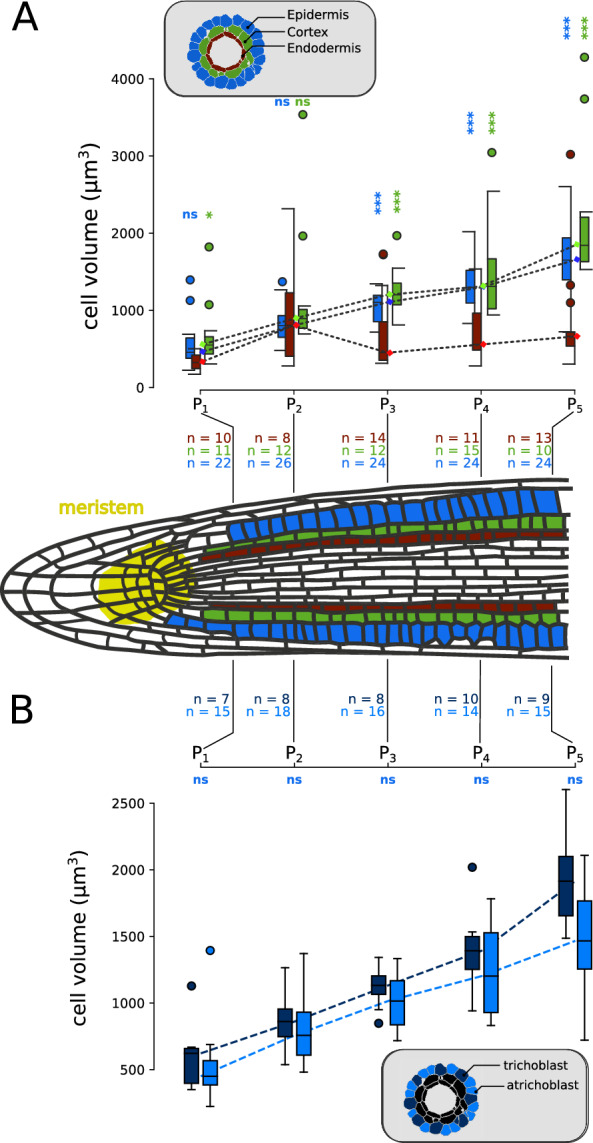
Longitudinal evolution along the root axis of cell volume, per tissue (**A**) and per cell type (**B**).** A** Cell volume in the three first root layers at five equidistant positions. Kolmogorov-Smirnov tests applied between the endodermis and either the epidermis (blue) or the cortex (green): * ($P<0.05$) and *** ($P<0.001$).** B** Cell volume of trichoblasts and atrichoblasts at the five same positions. Kolmogorov-Smirnov tests applied between trichoblast and atrichoblast distributions showed no significant differences (ns)

Plant roots show a stereotyped radial organization all along their longitudinal axis [20, 21] (Fig. 5A,B). This organization is emerging from the root tip, where a group of stem cells in the root apical meristem continuously divides to participate in the root growth. The stem cells are organized radially to generate the tissue radial organization. Each stem cell in the root meristem is at the origin of a cell file in a tissue layer and ensures its longitudinal growth. The cells close to the root meristem are the youngest and the cell age increases with cell position along the longitudinal axis. Hence, in a fixed root, the longitudinal axis unveils the time scale. The need for tracking divisions is less relevant in the root, but other cell relations can be pertinent and easily determined by the experimentalist.

The root is regionalized depending on several factors. The longitudinal axis can be divided in several parts [22, 23]: there is firstly a dividing part at the root meristem, then an elongation zone where cells grow and then a maturation area where cells end their development to be fully functional. Along the radial axis, the concentric tissues have different functions (Fig. 5A). From outside to inside, there are, firstly, the external cells that constitute the epidermis, forming a load-bearing barrier resisting the growth exerted by inner tissues, then cells forming the cortex, a storage tissue. The third layer of cells is the endodermis, a filtering tissue protecting the inner tissues composed of the pericycle (giving rise to lateral roots) and of the vascular tissues.

Given the root organization along two axes, one can ask how the different layers (radial axis) behave during development (longitudinal axis). Most of the developmental studies about the root have been performed in 2D, although recently, 3D measurements have gained interest [24–26] to phenotype more precisely cell events depending on various parameters (position, function, etc). With TreeJ, we can build metadata to analyze groups of cells by their type and by their age.

We selected five equidistant positions from the meristematic zone along a 3D segmented root image (the protocol for image acquisition and segmentation was the same as for the embryo). At each position, the cells from each tissue layer (epidermis, cortex or endodermis) were grouped into a separate tree, annotated by the corresponding position and layer (Fig. 5C). Here, we took advantage of the possibility in TreeJ to store into a single representation an arbitrary number of disjoint trees (Fig. 5D). Note that, in this application, cells were linked and incorporated in an arbitrary order in the trees without considering their actual lineage relationships, since the trees were used as a way to group cells by position and by tissue. Using the tag-based image creation functionality of TreeJ, a separated image was generated for each tree to extract measurements (Fig. 5E). The mean cell volume per group (position+layer) was calculated from cell volume measurements obtained with the Analyze Regions 3D plugin of the MorphoLibJ package [16] (Fig. 6A). The statistical analysis showed a similar average cell volume increase for the epidermis and for the cortex layer (Kolmogorov-Smirnov (KS) test, $P>0.05$). The endodermis contained smaller cells (KS-test; $P<0.05$ from Position 3) due to asymmetric formative division giving rise to endodermis and cortical cells [27]. These observations are consistent with a recently reported 3D analysis [24].

The epidermis is composed of two types of cells: the trichoblasts (giving root hairs) and the atrichoblasts [28, 29]. Previous 2D descriptions showed a differential shape between these two cell types [30]. As trichoblasts are wider and atrichoblasts are more elongated, we used TreeJ to evaluate whether their 3D volumes differed at the root tip. We discriminated the two cell types by their cell spatial position relatively to cortex cells and manually grouped cells from each type with TreeJ. As above, the cells linked and tagged at each position could easily be separated in two distinct images and the two cell types could be analyzed separately, a process we automatized over the five longitudinal positions with a python script. In spite of a slight systematic offset between the volumes of trichoblasts and atrichoblasts, with an apparent increase along the longitudinal axis, we observed no significant difference (Fig. 6B). This result is consistent with recent 3D observations [24] but further analyses, including larger samples, may be required to examine a possible growth divergence at the end of the analyzed region. TreeJ will be useful for this purpose.

With these applications, we have shown that TreeJ offers the possibility to store any cell relationships contained in a static image in a unique file. This file can be used outside of ImageJ/Fiji and it provides an easy access to the experimentalist’s annotations.

## Conclusion

With TreeJ, cell lineages can easily be constructed, visualized, and exported from a static segmented image. The formatted tree representation shows the relative division rates and can be used as a phenotype of the system to compare between individuals or mutants. Using TreeJ adds robustness to the lineage reconstruction process from static images and provides standardized representations that facilitate communication and sharing among the community. In addition to cell lineage reconstruction, TreeJ supports further analyses related to cell genealogy. Thanks to the TreeJ image export functionality, it is possible to extract the descendants from a common precursor or to rewind cell organizations back to previous configurations. Many analyses downstream tree reconstruction can be performed within the ImageJ/Fiji ecosystem. However, to perform new measurements or analyses, the reconstructed trees can be extracted and analyzed with, for example, dedicated python scripts. TreeJ can be used on any segmented tissue, be it animal or plant.

Future developments encompass the automation of pairing [31] or annotation [32] based on previous entries with machine-learning schemes. AI models could be trained with reference data sets created from TreeJ outputs. Adding a pretrained model in an ImageJ/Fiji plugin is now within reach [33–35].

## Data Availability

Most of the plant materials and the raw data of the figures used in this study are available on the Github web page at https://github.com/L-EL/TreeJ/. The plugin is also available at http://imagej.net/plugins/treej. Version v1.0.0 can be found here [36]. Project name: TreeJ. Project home page: https://github.com/L-EL/TreeJ. Archived version: 10.5281/zenodo.10041643. Operating system(s): Platform independent. Programming language: Java. Other requirements: java 1.8 (ImageJ2/Fiji 1.51n or higher) and itextpdf plugin. License: GNU GPL.
